# Implementation of Safety Planning at 988 Crisis Centers in Missouri: A Mixed Methods Study

**DOI:** 10.21203/rs.3.rs-7925708/v1

**Published:** 2025-11-18

**Authors:** Morgan C. Shields, Danielle R. Adams, Theresa Anasti, Gabriela Musickant, Zohra Kantawala, Rachana Cheu, Ryan Lindsay, Byron J. Powell, Jonathan Purtle

**Affiliations:** Washington University in St. Louis; University of Missouri; Washington University in St. Louis; Washington University in St. Louis; Washington University in St. Louis; Washington University in St. Louis; Washington University in St. Louis; Washington University in St. Louis; New York University

## Abstract

**Background:**

The United States faces a growing behavioral health crisis. To improve access to crisis services, the federal government launched the easy-to-remember number, 9-8-8, for the National Suicide Prevention Lifeline in July 2022. However, little is known about the quality of care provided through 988 crisis lines, particularly regarding the use of evidence-based practices, such as Safety Planning. This study examined variation in employee attitudes toward Safety Planning, how it is operationalized, and factors that influence its implementation on crisis lines in Missouri.

**Methods:**

We used a convergent triangulation mixed methods design. A survey assessed demographics and attitudes toward Safety Planning using the Intervention Appropriateness Measure (IAM), Feasibility of Intervention Measure (FIM), and Acceptability of Intervention Measure (AIM). Semi-structured interviews explored staff’ experiences in depth. Regression models identified predictors of attitudinal measures, and qualitative data were analyzed using the Twenty-First-Century flexible coding approach, integrating deductive and inductive methods.

**Results:**

Of the 97 respondents, 79.4% were frontline crisis counselors and 20.6% were in leadership positions. In quantitative analyses, average scores on the IAM, FIM, and AIM were all high; however, graduate-level education was negatively associated with scores. Interviews (n = 28) revealed overall strong support for Safety Planning. The synthesis of these interviews resulted in four themes, including (1) counselor’s attitudes, (2) caller characteristics, (3) caller-centered practices, and (4) quality assurance practices and culture. Most barriers and facilitators to Safety Planning were related to the social, economic, and health circumstances surrounding callers; organizational supports (e.g., culture, training, quality monitoring, feedback) were important drivers.

**Conclusions:**

Safety Planning is viewed positively by 988 crisis line staff in Missouri, which is facilitated by organizational supports; however, there are barriers to its utility and impact, such as caller circumstances and the brief, phone-based structure of care. To our knowledge, this is one of the first empirical examinations to understand the use of Safety Planning at 988 crisis centers. Findings underscore the need to refine conceptualizations of “quality” in crisis services, to examine how evidence-based practices like Safety Planning vary within and across 988 centers, and to identify meaningful, appropriate, and feasible accountability metrics.

## Introduction

The United States is in a behavioral health crisis, with suicide rates, substance use, and mental health conditions among youth significantly increasing over the past decade (1). While over one in five Americans experience a behavioral health disorder in a given year, less than half access care, a number that is further reduced for marginalized populations such as racial and ethnic minoritized people (2,3). The U.S. Substance Abuse and Mental Health Services Administration defines a behavioral health crisis as: “A disruption in a person’s thoughts, emotions, behaviors, or functioning that leads to an urgent need for assessment and treatment to prevent the condition from worsening or becoming dangerous,” such as resulting in a suicide attempt (4). To strengthen the U.S. crisis behavioral health response systems, the federal government aimed to improve access to crisis lines by replacing the 10-digit National Suicide Prevention Lifeline with the easy-to-remember number, 9-8-8 and expanding the name of the line to the 988 Suicide and Crisis Lifeline (5). 988 launched in July 2022 has been contacted 17.7 million times since its launch and 8.3 million times between June 2024-July 2025 (6,7). This national 988 initiative resulted in an influx of federal, state, and local funding directed toward crisis lines, along with increased requirements related to quality standards for line (8,9). At some 988 call centers, these standards include the use of Safety Planning, an evidence-based intervention for suicide prevention (9,10). As such, the implementation of the standardized 988 lifeline and associated policies and regulations may affect not only access to crisis care but also the quality of such care.

Safety Planning is a collaborative process between a counselor and client, whereby the client is supported in creating a series of action steps to respond to suicidal ideation to prevent suicide attempts. While Safety Planning is primarily used for suicide prevention, it has also been adapted for other mental health crises, such as self-harm and other associated emotional states (11). A manualized version of Safety Planning outlines six Safety Planning protective factors to identify: 1) warning signs of triggers that indicate suicidal ideation is likely to occur, 2) internal coping strategies, 3) social contacts or social locations for further distraction, 4) supportive contacts who can provide further assistance, 5) emergency resources, and 6) steps to ensure the safety of the home environment and minimize the patient’s ability to act on suicidal urges (12). In addition to its proven efficacy in suicide prevention, Safety Planning has multiple strengths, including its efficiency as a standalone intervention, and ease of implementation (13).

While Safety Planning is adaptable to various settings and populations, spanning emergent and non-emergent environments (14–16), the extant evidence for Safety Planning is focused primarily on contexts of established clinical relationships, such as in psychotherapy, and specifically concerning suicidality (15–17). While there has been some evidence for the value of Safety Planning in emergency settings, such as inpatient psychiatric facilities and emergency departments, evidence in the context of crisis lines such as 988 is lacking (16,18,19). Given the unique context of crisis lines (e.g., lack of face-to-face contact, very brief encounters, lack of established relationship), there are likely distinct barriers and facilitators to the use of Safety Planning in this setting, particularly in the context of evolving 988 quality standards and contractual requirements (20). Moreover, the appropriateness of Safety Planning might vary across callers based on clinical needs (e.g., substance use) and social needs (e.g., availability of social supports, access to healthcare), which are often difficult to address with a single, brief intervention. While the range of empirical questions that can be asked related to this topic are diverse (e.g., effect of specific implementation strategies, impacts of Safety Planning on outcomes, disparities in the use of Safety Planning), we were interested in taking a more exploratory approach to understand how Safety Planning is currently being used in 988 crisis centers and the experience of counselors with its use.

The current study examined how Safety Planning is used at crisis centers handling 988 calls in Missouri, focusing on the perspectives of frontline crisis-line counselors and center leadership. Missouri was recently recognized as a national leader in 988 access metrics, including answer rate and response times (21). Specifically, we use a mixed methods approach to understand variation in staff attitudes towards Safety Planning, the ways in which Safety Planning is operationalized on the crisis line, and the factors that influence the ease with which counselors can use Safety Planning and the scope of its impact.

## Methods

### Study Design and Participants

We used a convergent triangulation mixed methods design (22). Researchers administered an online survey to staff at all six 988-participating centers in Missouri from July-November 2023. We asked senior leadership at centers to send out an email to all relevant staff explaining the study and inviting them to participate by filling out a brief survey. To lessen the burden on organizations and to protect the identity of participants, we did not request specific email lists and the link sent was a general link, rather than personalized to each potential employee. A consequence of this approach is that we do not know the ratio of respondents to eligible participants. Staff interviewed included 988 managers (20) and frontline crisis workers (77). The survey gathered demographic and professional characteristics of the respondents and included several measures of staff attitudes towards Safety Planning. Towards the end of the survey, participants were able to indicate their willingness to participate in a 60-minute semi-structured interview; the research team followed up with interested individuals to schedule an interview. This design allowed us to understand employee-level professional characteristics associated with attitudes towards Safety Planning using quantitative measures, while the in-depth qualitative interviews allowed us to gather a more nuanced understanding of the use of Safety Planning, including factors that influence the ways in which it is used and its impact.

### Survey Measures

#### Staff Characteristics.

The survey included the following categorical demographic variables: age (18–34, 35–54, 55 and over), gender (male, female, non-binary), race (White, Asian, Black or African American, Native Hawaiian or Other Pacific Islander and American Indian or Alaska Native), ethnicity (Non-Hispanic/Latino/a/x and Hispanic/Latino/a/x) and the following professional characteristics: education (4-year college degree only, graduate-level degree), role in the organization (frontline crisis counselor, leadership), years of professional experience (0–1, 2–4, 5–10, over 10 years), years in current role (0–1, 2–4, 5–10, over 10 years), having a clinical license to practice in the state (yes, in the process, no), full-time employment at the organization (yes, no). A summary of demographic information is in Table 1

#### Outcomes.

We measured workers’ self-reported attitudes toward Safety Planning using measures of implementation outcomes that have strong psychometric evidence, including the Intervention Appropriateness Measure (IAM), the Feasibility of Intervention Measure (FIM), and the Acceptability of Intervention Measure (AIM) (23,24). Each measure was adapted to use Safety Planning as the intervention of interest and included four items on a 5-point Likert scale, with 5 representing the highest level of appropriateness/feasibility/acceptability (see Appendix A for questions).

### Qualitative Interview Guides

The Consolidated Framework for Implementation Research (CFIR, e.g., outer and inner-setting factors) informed the interview guides for the semi-structured interviews (25). We used the CFIR to guide specific domains that we probed during the interviews along dimensions of employee characteristics and attitudes towards 988 in general and Safety Planning specifically, characteristics of Safety Planning as an intervention shape the experience of its use, inner-setting factors that shape the functioning of 988 and use of Safety Planning, outer-setting factors (e.g., policy) that influence the impact of 988 and the nature of Safety Planning use, and caller characteristics that modify the use and impact of Safety Planning. See Appendix B for a copy of the interview guide. These interviews enabled us to understand more deeply staffs’ attitudes towards Safety Planning and relevant factors that impact its use or modify its impact.

### Analyses

As this was a convergent triangulation design, quantitative and qualitative data were analyzed concurrently, meaning the quantitative and qualitative aspects of the study were analyzed independently of each other.

#### Quantitative.

For quantitative analyses, the authors examined univariate statistics and then fit three multiple regression models predicting IAM, FIM, and AIM scores with staff professional characteristics as predictors, including clustered errors and a random intercept for the organization in all three models. Stata (version 18.5) was used for all quantitative analyses.

#### Qualitative.

Qualitative coding followed the Twenty-First-Century flexible approach, combining deductive and inductive methods of analysis.^18^ This coding analysis approach captures concepts relating to extant literature and new insights. Moreover, it facilitates analysis that uses qualitative data analysis software in large-scale qualitative data sets. The CFIR formed the basis of the initial set of broad-based, deductive codes, and the research team refined and created new codes throughout coding, using an iterative process. Six researchers independently coded interviews and met weekly to continuously compare themes, patterns, and concepts found in the data to develop a code book. These weekly meetings occurred for several months until a comprehensive codebook was established, and agreement was reached between the team. In the next stage of analysis, the codebook was used to code each interview, each being reviewed by two separate coders. Coding teams met weekly to discuss discrepancies in the codebook application and identify the need for codebook refinement. In the last stage of analysis, codes were synthesized into themes. While the interviews covered a variety of categories, we focused analysis for this paper specifically on insights related to Safety Planning.

## Results

### Quantitative Findings

Table 1 describes sample demographics across survey and interview samples. There were 97 respondents to the survey. Of the 97 respondents, 79.4% (n = 77) were frontline crisis counselors, and 20.6% (n = 20) were in leadership positions. Of the total sample, 72% (n = 70) had a graduate degree, about 46% were younger than 35, about a third had one or fewer years of professional experience, about 42% (n = 41) were in their current role for one year or less, about a third (n = 6) had a clinical license, and 60.8% (n = 59) were full-time staff.

Average scores on measures of staff attitudes towards Safety Planning – the IAM, FIM, and AIM – were high (average mean ≥ 4.3 on a 5-point scale). Table 2 reports the results from the regression models. Graduate-level education was the only significant predictor of all three measures of attitudes toward Safety Planning. Having a graduate-level education was associated with a 0.36-unit decrease in intervention appropriateness (IAM,) a 0.35-unit decrease in intervention feasibility (FIM), and a 0.49-unit decrease in intervention acceptability (AIM).

### Qualitative Findings

About 30% (n = 28) of survey respondents participated in the interview portion of the study (Table 1). Findings from the interviews indicated overall strong support for Safety Planning, though participants provided nuanced insights into the various factors that shape their experience of using Safety Planning. The synthesis of these interviews resulted in four overall themes, including (1) counselor’s attitudes, (2) caller characteristics, (3) caller-centered practices, and (4) quality assurance practices and culture. We describe these four themes below.

#### Counselor’s Attitudes.

Participants largely viewed Safety Planning as being valuable to callers, despite limitations (which are described in subsequent themes). Participants expressed positive attitudes towards Safety Planning:

“I am a fan of safety planning. And I think that if you really could get an ideal call and you really could sit and walk through all of it, that would be fantastic.”

(C3)

Factors that contributed to counselors’ positive view of Safety Planning included the ease with which it was implemented and supported within their organization, as evidenced by a participant who shared:

“I think it’s great. I think it is super useful for every caller and every person, even if it’s not as in-depth with someone who hasn’t had ideation or anything like that. I think it was rolled out well. I think it’s pretty clearly understandable to anybody, even if they don’t have a history with their mental health or mental health professionals. Yeah. It works for me. It works for callers. Yeah.”

(C19)

Participants found Safety Planning components to be pragmatic, with Safety Planning helping to structure conversations with callers, but that it is important that it only be used as a guide and that counselors be flexible and adapt to caller’s needs. This is illustrated in this participant’s description of their orientation to Safety Planning as an intervention:

“I think that the specific structure of it … is good as a guide, but you can’t just stick to it. I like to use it in a flexible way. Where is it written down? Is it not written down? Sure, written down is better, but then also, I don’t force all my callers to write it down or stress that part. Sometimes it’s very conversational, sometimes it’s planting a seed in their mind that what you got planned for later. So, I like it. And I also like to use it flexibly.”

(C5)

Caller characteristics moderated impact, as described in the caller characteristics section below. However, even in “ideal” circumstances, participants described their own process of “making peace” with the limited scope of impact a crisis line can have for both individuals and the larger population. The lack of face-to-face contact, for example, was described as a barrier to connection:

“Sometimes safety planning over the phone just sucks. I mean, people aren’t as connected. I think that that’s a huge barrier.”

(C31)

Another described the psychological impact that comes with recognizing that the 988 crisis line is limited in how much it can improve population mental health:

“I’d say the two hardest parts for me personally… one, is not having resolution on certain calls. I’ve had some fatal and near fatal calls and I was lucky enough to get resolution. However, most times that doesn’t happen and we really just don’t know. The other thing is reading statistics for me is really hard because it’s like how we’re barely making a dent. It’s like there’s so many people that don’t call.”

(C19)

One participant noted how they tailor their approach given the limitations of Safety Planning over the 988 line:

“I think that all of us probably had our favorite things out of a safety plan that we would pull out depending on the situation. With some folks, that’s going to be really pushing professional supports because you can tell they’ve got years of trauma. I’m not going to do much on a 15-minute phone call. Even a 50-minute phone call, I’m not going to be able to do much. Some of it, you really spend your entire call trying to break down barriers toward one segment of a safety plan.”

(C3)

#### Caller Characteristics.

Staff often spoke about caller characteristics as a key factor that impacts Safety Planning’s use, fit, and effectiveness; indeed, many barriers to Safety Planning were described through the lens of caller characteristics. While we refer to these as caller characteristics for the purposes of naming a collective theme, it is important to distinguish that some of these characteristics are not inherent to the caller but are rather reflective of a caller’s position in a larger ecosystem, with factors outside the caller shaping what they bring with them to the crisis line.

Important caller characteristics associated with ease of Safety Planning from the staff perspective spanned caller attitudes, past experiences with crisis services, expectations, and general demeanor (e.g., degree of openness, trust, hostility, agreeableness). For example, one participant noted how “familiar callers” (those who call regularly) often call for connection, not necessarily because they are experiencing acute crisis:

“Most frequently, they’re not presenting as having any ideation. Some of them use the line almost like we’re their friends and they’re calling for updates to let us know how they’re doing.”

(C19)

Participants expressed frustration when engaging with familiar callers whose needs go beyond what the 988 line is designed to address, noting that these individuals often find neither the crisis line nor community resources helpful—which reflects broader issues with how staff are trained and supported.

“There’s one client in particular that, when we see her name coming up, we kind of just all groan because we know what’s going to happen. We’re going to hear the trauma history. We’re going to hear how therapy doesn’t work. We’ll hear about how breathing doesn’t help. And with her, a lot of it’s still reminding her that she is choosing to not do these things. And with her, we do take a little bit of a different approach because she’s not suicidal.”

(C31)

Such experiences illustrate how structural inadequacies in community-based mental health and social support systems leave crisis counselors addressing needs that far exceed the intended scope of the 988 line, underscoring the importance of training that better prepares staff to prevent potential harm to callers. However, this quote also illustrates negative attitudes staff have towards some callers, that might come from a place of bias.

The role of callers’ disposition and history is exemplified in the following quote:

“You work with a lot of folks who say, “I’ve tried everything. It doesn’t work. It doesn’t work.” So really resistance to even considering that there might be something that could help them in the moment. Emotional dysregulation is such a big part of the calls that we would get. People are just very, very dysregulated a lot of times when they call us. So, to even try to get them to think through and cooperate… I’m not trying to say that in a disparaging way, but just if folks have been depressed for 20 years, they say, “I’ve tried everything. Nothing helps,” sometimes they’re just very resistant to even the idea of things that we’re changing.”

(C3)

At times, callers have a misunderstanding of what the 988 line is intended for:

“I get a caller the other day and was like, ‘Hey, my brother is in a hospital, can you look him up for me because it’s mental health related?”

(C5)

And fear of carceral interventions, such as police intervention and involuntary hospitalization, can be triggered by certain components of Safety Planning:

“…when you start to address things like access to lethal means, they may be, “Oh, no, no, it’s fine. I’m perfectly fine.” Or once you start to get into something a little bit more structured, like a safety plan, that can make people feel very nervous, especially the last step of our safety plan, which is emergency procedures … A lot of people hear ‘emergency’ and they hear hospital, and that doesn’t have to be the case.”

(C41)

Participants described the complexity of callers’ situations as limiting the scope of impact that Safety Planning could have, which were situations that required large structural interventions (e.g., employment, housing, transportation, insurance):

“I guess the stuff that’s challenging would be maybe that it’s really difficult to help people whose life circumstances are so bad that it doesn’t seem like what I can do would make that much of a difference.”

(C28)

For example, one participant noted:

“I mean, a lot of people who are calling in, they don’t really have any supports. And as I’m sure we both know, access to mental health care isn’t the greatest, especially for people in rural areas, people of low socioeconomic status, all of that. And so, I think that lived experience as well as just me having done this for a bit kind of makes me more aware of the limits of safety planning too.”

(C51)

The unique nature of a person’s mental health crisis and life journey might not be the best fit for Safety Planning, such as when parents call needing support for their adult children experiencing psychosis:

“It’s hard for me to teach a mother, a 68-year-old mother about coping skills for her nearly 50-year-old son… Teaching them coping skills to teach her son, who’s going through acute psychosis. I can’t teach mom everything there is to know about psychosis and how to recognize signs, especially not when she’s fearing for her safety. And so, coping skills for the family. Who can we call? How can we handle someone in crisis? So I think our safety plans could be a little bit better adapted towards that, perhaps even talking about substance use. Right now, we assess for it, we talk about it, “Are you under the influence?” But that’s about it.”

(C41)

The extent to which a caller had people in their lives whom they could reach out to for support, beyond formal services, was also a factor that shaped the impact of Safety Planning. Indeed, a component of Safety Planning includes creating a list of people one could reach out to when in crisis; the absence of a formal and informal support system challenges the Safety Planning process:

“A lot of these people have no one. So part of the safety plan is people they can reach out to and they don’t have any support system. And that’s a big component of the safety plan, being able to reach out to others because we’re human and that connection is needed to reach out to others when you’re needing others.”

(C52)

For minors, this introduced unique constraints:

“If it’s a minor, we have to get some sort of trusted adult involved and a lot of times the trusted adult will be a part of the presenting problem”

(C51)

Situations that involve intimate partner violence (IPV) might similarly exacerbate these limitations, as exemplified in this quote:

“Safety planning is tricky because …either they don’t have professional supports or they don’t have the ability because their partner has control, or when it comes to friends and family, where they have been isolated, they have been all the things that happen with IPV that make those things difficult…More often than not, it’s finding a safe place to stay, aftercare resources, things like that, as opposed to personal supports, like social supports.”

(C19)

Similarly, respondents noted that some callers lacked insight into their triggers and/or struggled with a sense of self-efficacy over their problem or found suggested coping strategies to be ineffective, which can serve as barriers to Safety Planning, particularly during such a short period of engagement on the call.

“I guess first thing that comes to mind would be if the caller doesn’t think they’re capable of solving their own problems, or at least, I’m not sure of the right word, caring for themselves or managing a crisis.” “Another thing that can get in the way is when people are like, “Well, but I already tried that, but it didn’t work, so I called you.” So that’s a compelling counterargument, hard to argue with it if they tried stuff and it didn’t work. And then I guess you try to come up with other things or search more deeply for things that they’ve tried in the past or haven’t tried in a while. So, certainly, if somebody is dealing with something like depression, their motivation level can be pretty low. So, if they’re an artist or they write in their spare time, trying to do a painting or writing a poem might feel a little bit daunting at the time.”

(C28)

Finally, participants described some behavioral factors related to a caller’s capacity to engage, which included whether they were intoxicated, extreme psychological states that challenged interpersonal connection, and general engagement with the process. For example, one participant remarked:

“…if they’re intoxicated, that obviously makes it a lot harder to safety plan … There’s a lot of times where people will just abruptly disconnect the call before the conversation’s fully done … if they’re not reachable back then there isn’t really a solid safety plan because you can’t reach them again.”

(C51)

Or general past lack of interest in Safety Planning:

“Sometimes they’ll shake it off. Sometimes they’re like, “Okay,” and they’ll go over a couple of the skills. But most times, they’re not really calling for that and they already have one. A lot of times they’re like, “I don’t need to review it.” That makes it pretty difficult. Yeah.”

(C19)

#### Caller-Centered Practices.

There was a strong and consistent emphasis placed on caller-centered practices, with caller engagement in Safety Planning being key to its relevance:

“You don’t want to make up what works for you because you’re not that person. You want to definitely get their input and you want them to be the facilitator on that safety plan. It’s important to make sure that you are getting their words and what works for them, so that you know it works.”

(C9)

Participants described the importance of tailoring Safety Planning (i.e., selecting safety planning strategies that build on existing capacities and assets so that the plan feels realistic, empowering, and actionable) to each caller’s presentation or needs, and that the nature of Safety Planning allowed for this flexibility. Sometimes tailoring takes the form of casual, unstructured conversation that allows for certain components of Safety Planning to naturally emerge without labeling the conversation as such. Participants described re-phrasing and integrating questions in ways that were more conversational and responsive to caller’s concerns and prior experiences, and in encouraging callers to consider what works for them rather than generic coping strategies, as evidenced by one participant who shared:

“So if somebody’s biggest buffer is like a pet, then safety planning would probably involve, “Does it calm you down a little bit if you hold your pet for a little bit?” Another participant shared how they tailor Safety Planning to ensure it will be relevant to their caller: “I’ll say, ‘Well, what works for you?’ That’s generally how I put… ‘What are some coping skills that help you calm down.’ And I add what works for you because they can rattle off some coping skills, but it’s something they don’t use. And I want them to have more ownership on it.”

(C5)

Participants also adapt Safety Planning to consider the ecological context of the caller:

“I always tend to adapt them because everyone is different, and it has to be collaborative. Meaning I have to make it work for you, and I have to be very creative. If you are in rural Missouri and you do not have access to a crisis center, that is not an option for you. If you are in a violent relationship and you don’t have any supports, then your external resource is going to be very different.”

(C41)

While some participants noted personal comfort with tailoring safety planning to meet the needs of callers, some noted that not all their coworkers are as adaptable when implementing the intervention:

“And I think that’s what’s really frustrating for me is that people have no desire to adjust as needed to meet clients’ needs and are just doing things based on the template and not even thinking about it critically and are just like, ‘I’m going to word this as it is, and if it doesn’t go well, that’s not my fault,’ kind of thing. I don’t agree with that. I think talking it out with other people, coworkers that I trust, and being like, ‘What do you think about this?”

(L17 / C17)

Indeed, participants mentioned that some callers found the language of Safety Planning to be off-putting because it came across as too clinical and distancing or because prior experiences of Safety Planning were too focused on the plan and not the relationship, creating a context for relational disconnection. For example, one participant described the language and transition to Safety Planning as being a challenge:

“When I have mentioned the words before, when I am doing the head-on approach of ‘we can build a safety plan together,’ things like that… I do feel people back away a little bit more when they hear that, even if they still go on with it. It’s a hard transition. I see where the structure of the conversation gets to the point of the safety plan, and it’s ordered in that way for a reason, and things like that, but I think that sometimes I feel like people tighten up when they hear it. I don’t know that it’s the word. I think it might be the transition into it.”

(C19)

Participants also discussed that while they work to be caller-centered, they also face barriers around the limited amount of time each call is allotted, as evidenced by this participant’s quote:

“I would say trying to meet people where they are, provide them with what they want, at the same time, balancing the time length of the call and the focus of the call. I may be focused on safety, while some people are focused on wanting to vent. And so being able to provide that, so not making them feel like they’re being rushed or pushed away. Even though we’re a free resource, we’re a limited resource. And so being able to utilize that in an appropriate way is always a balance with people.”

(C41)

As part of being caller-centered, participants placed considerable value on ensuring the least restrictive intervention was deployed:

“Our policy was, I thought, really, really good as far as it was a really last resort for us. But I would say that there’s definitely some stigma and some bias about we’re always looking to call police. Absolutely not. We want to do everything but.”

(C3)

Participants consistently recognized that sending police to check on a caller or transporting a caller to the hospital comes with risks and could traumatize callers, and that callers had distrust towards these interventions due to their own experiences or word of mouth, as evidenced by this comment from a participant on the topic of calling the police for wellness checks:

“Ultimately, for a million reasons, we don’t want to have to do it. It’s just really, it can be very invasive and just very violating and dangerous, downright dangerous for people of different cultures as well. That’s really big. The fear is there. I think the word of mouth also spreads like wildfire.”

(C19)

Though some participants noted contradictions in their trainings and awareness of the harms that come from carceral interventions and the organizational policies that require inclusion of these interventions in Safety Plans:

“We’re trained on cultural considerations regarding contacting the police for particularly racial considerations, as well as people with a trauma history. And then, going to the hospital for people with a trauma history related to mental health or any medical trauma, whatever. But we’re also required to encourage people to review emergency procedures and that are, call 911 or go to the emergency department. So, I feel like that doesn’t cover all clients. So, I don’t know, that seems counterintuitive sometimes with what we’re trained on and what we’re supposed to do.”

(C46)

As part of being caller-centered, most participants described procedures for following up with callers when possible and connecting them with resources in the community. Nevertheless, participants described caller characteristics that served as barriers to avoiding the least-restrictive intervention and to providing caller-centered support, such as intoxication, lack of engagement, time limits on calls, or when supporting third-party callers (e.g., community members who might witness someone acting erratically in public).

#### Quality Assurance Processes & Culture.

Participants highlighted the important role that their organizational structures, culture, and leadership had on their comfort and skill in using Safety Planning. A central component of this was robust supervision. Participants emphasized the importance of maintaining transparent and holistic relationships with their supervisors. These relationships fostered environments where participants felt respected and heard, and that allowed frontline experiences to directly improve the organization’s functions, as expressed in the following quote:

“My supervisors, I have felt like I can have a super transparent relationship with. We’re actually able to communicate in a way that’s like, “Hey. Also, life and how this job is affecting me, and how do I cope with that, and how am I feeling about the equity in the organization,” and things like that. I feel like I’ve been able to have really transparent conversations, and I feel heard. I genuinely feel heard because there have been things that I’ve brought up before that were like, ‘Oh, actually, yeah, maybe we should pay attention to that.’ That’s happened with other employees, too, or other people have also said, ‘Come in with fresh eyes’ and said, ‘Hey. I see this thing that might be able to be done better.’ They’re like, ‘Oh, actually. Yeah. That makes total sense.’ They’re really reactive as well and proactive.”

(C19)

Participants appreciated supervisors providing them with autonomy in their work, which allowed for their use of (26) in adapting Safety Planning in a way that was acceptable and feasible for the situation. By allowing frontline counselors the leeway to interpret ambiguous situations in a way that they thought was best, supervisors were seen as supporting the expertise of frontline counselors. Supervisors were also described as supporting participants’ mental health and ability to compartmentalize:

“To have it from micro scale to macro is really great. Anytime that I’ve needed, whether it’s been an insurance problem… which happened, we had a little blip. But whether it’s an insurance problem or something with scheduling or anything like that, you’re constantly told like, “If you need anything, just text me,” even if I’m not on call. My supervisor, actually, when I had my near-fatal and fatal calls, it was during the holidays, and she was in her car with sick kids, and she called me to check in and see how I was doing. Everyone is very in tune. Anything that needs to be run up the flag post is pretty immediate, from the mentors who are very much micro on the floor with us to the supervisors who have the ability to actually make the changes and do things.”

(C19)

“…Because we had an alias that we had to use on the lines. He said, “When you come in here, you are your alias. And when you leave, you’re not. You leave your alias there.” Boy, I think that was super, super helpful for me. I kept that mindset the entire time I was there.”

(C03)

Rigorous training and continuing education opportunities were also described as central to participants’ ability to manage calls and to use Safety Planning:

“I’ve never had more applicable, thorough, relatable, eye-opening, timely training in a job before in my life. That comes from, we have the national portal where we have the Lifeline National sponsored training, and then we also have our internal local specific training as well, which ranges a bunch of different topics.”

(C19)

These trainings are also standardized and facilitated by Vibrant, the administrator of the Lifeline National:

“The Lifeline National helps structure the calls and the ways to engage with people and treat people in different circumstances and stuff like that, and [their trainings] remind you of what a crisis is and what it could look like to anyone. Also, managing your own expectations and thoughts about the job. It’s all very relatable and we have monthly trainings, so it’s ongoing constantly.”

(C19)

Participants described trainings that included specialized topics and populations:

We have had everything … from minors at risk, gender-affirming mental healthcare. I’m trying to think of some of the other ones. Just in time training for, which is what it’s called, but for disaster survivors and responders. We’ve had training to help people with different physical abilities. Oh, gosh. People of different cultures in terms of religions and race and things like, and how to help them if you don’t have the same background, a variety of topics.”

(C19)

Supervision also included regular quality monitoring processes, such as silent listening to calls and audit and feedback. One participant noted how these practices act as a facilitator towards their use of Safety Planning:

“Our safety plans, our documents, are routinely reviewed. Our live calls are reviewed. We receive regular reviews, as well as when you first start, you have a 90-day probation period where all your calls and documents are reviewed, and then it becomes your high lethality calls are reviewed. And then you have weekly check-ins where you go over your case reviews with your supervisor. So, I do that on a weekly basis, where we go over how I handled the call, the safety plans. We also have a built-in policy related to consulting before you contact any emergency services or reach out to something like that. We always encourage people to consult calls, so we can talk about how well did they safety plan? Have we done everything to safety plan with the client before we’ve contacted emergency medical services or the police department?”

(C41)

Participants also emphasized the importance of lateral support from their colleagues:

“We have a Zoom chat so if I get stuck … And I mean, we’re all kind of aware of what’s going on around us throughout the day. So, if we hear someone talking to someone in safety planning and they’re suggesting coping skills, I mean, we’ll kind of roll our chair back and look at them and be like [gestures]. That means bubble blowing. Bubble blowing can be a deep breathing coping skill. And just reminding each other of things because I mean, while we’re dealing with people in crisis, we all lose our train of thought and we all kind of forget other coping skills sometimes. And we all have certain ones that are our go-to diehard coping skills. And so, being able to remind each other, I think that that’s definitely helpful.”

(C31)

While participants largely described their organizations in a positive light, there were some critical comments made about the level of support offered by some of the organizations in our sample. This is described well in the following quote, which critiqued their organization’s emphasis on measurable performance and efficiency, rather than the human experience, which they describe as affecting morale:

“… not having enough people to take the calls and getting different answers on topics. And it really affects morale, and we could blame it on the fact that maybe the budget isn’t enough to have enough managers to be able to manage all the things that we need to have managed. And there’s also not enough for frontline staff so that other call takers aren’t getting burnt out. But management has been more reactive, especially with changes… You know when something gets accredited and then something starts to change, right? You’re gunning for accreditation or something, and we’re looking for proof of something. I’ve worked in some contexts where I’ve tried to help organizations get accredited as well, and there’s a part of it that feels performative for me. I used to work in a corporate environment as well, so I can detect these kinds of KPI [key performance indicator] approaches that I’m like, we’re not centering on the human experience of what we’re doing here. We’re not like a call center for, I don’t know, The General car insurance. There are reactive business-oriented changes that are par for the course, but at the same time really hurts morale.

I had my own experience where I was just like, oh, I feel replaceable. I feel baited and switched. I don’t feel invested in as a long-term employee. This is not a sustainable long-term option for me. You don’t feel invested in. And that’s a tough part. I wish our field had these secure union jobs that you could work the rest of your life, knowing that you could have a fulfilling job, and you could have a future where you didn’t have to work anymore. That’s one thing that I would love to see. But I see the reactions of management being more and more austere in that.”

(C37)

Overall, participants described the importance of their own commitment to the role and the initiative that they take to continuously learn about best practices and seek out support. This personal commitment was described as a buffer against lapses in management, allowing frontline workers to show up as clinicians while advocating for change within their organization to improve care for callers. One participant describes their strategy for approaching challenges with management in the following:

“I’ve also become outspoken in that I don’t trust my direct manager’s clinical approaches to things. He’ll give me wrong information most of the time, but I just go over his head if I need to really clarify something that’s important. At one point, I saw other supervisors giving advice to say that it’s okay to tell clients that there were call time limits. And I was like, ‘No.’ And then I asked my supervisor about that and he was like, ‘I’m not sure.’ And so, I went over his head and I was like, ‘What is up with this?’ And she was like, ‘Oh, no, that should not be happening.’ So, there is poor oversight when it comes to the management, and so I feel like a big part of it is clinicians and leads holding the management accountable to making sure that everyone is on the same page because it really makes it harder when, say, somebody calls in for the second time today and they didn’t get an adequate safety plan the first time.”

(L17 / C17)

## Discussion

Among crisis centers in Missouri, Safety Planning, an evidence-based practice for suicide prevention, has been established as a core component of the services provided to 988 callers, with strong support from frontline workers and organizational leadership of crisis centers. The enthusiasm for Safety Planning was reflected in results from the survey data as well as the qualitative interviews. The average scores on the quantitative measures of appropriateness, feasibility, and acceptability of Safety Planning were high, though workers with graduate-level education had slightly lower scores. Results from the semi-structured qualitative interviews suggested that workers had positive sentiments towards Safety Planning, but they also described a range of factors that shape the ease with which they could implement Safety Planning and the impact that Safety Planning might provide to each caller. To our knowledge, this is one of the first empirical examinations to understand the use of Safety Planning at 988 crisis centers.

Curiously, participants with graduate-level education scored Safety Planning appropriateness, feasibility, and acceptability as slightly lower; while interesting, this is not entirely surprising. One plausible explanation for this finding is that graduate-level counselors have a greater degree of knowledge and understanding regarding the types of robust interventions and supports callers might benefit from, leading to greater awareness of the limitations of brief Safety Planning interventions delivered over the phone. Another potential explanation is that some master’s-level counselors might be working at 988 crisis centers to gain hours to obtain licensure to practice as an independent clinician and might dislike the structured nature of the 988 process and Safety Planning. Moreover, while previous research suggests that individuals with higher levels of education, such as a master’s degree or higher, tend to have more positive attitudes toward evidence-based practices (27), there is wide variation in how people are trained in graduate programs, and not all programs embrace evidence-based practice (28,29). Nevertheless, while there was a statistically significant difference by education level, this difference was relatively small and might not translate into meaningful differences in actual engagement with Safety Planning, competency, and broader care quality.

The qualitative data helped illuminate the more nuanced perspectives of staff regarding the use of Safety Planning when speaking with 988 crisis line callers. Staff described broad adoption of Safety Planning and support from their organization through informal (e.g., lateral support from colleagues) and formal (audit-and-feedback mechanisms, quality reporting, supervision, continuing education) implementation strategies. Most barriers and facilitators to Safety Planning are related to caller characteristics and the social, economic, and health circumstances surrounding callers. Staff described some callers as having limited social supports or internal coping strategies, making it challenging to develop a viable Safety Plan. While Safety Planning is intended to be a flexible intervention that can be tailored to individual situations, the extent to which staff were able to implement Safety Planning processes was influenced by the callers’ engagement and their ability to generate responses to the Safety Planning questions. Sometimes, prior exposure to either Safety Planning or mental healthcare services was related to distrust among callers and affected the degree to which they felt Safety Planning was a beneficial use of their time with counselors. The time restriction on calls was reported as a significant barrier, especially for callers with reduced trust, more complex situations, or those who were generally more in need of robust interpersonal engagement. The inherent time constraints of crisis calls can prompt counselors to overemphasize problem-solving, leading them to offer quick solutions rather than prioritizing validation and collaborative safety planning. Indeed, while staff recognized the value of Safety Planning in addressing 988 calls, they also acknowledged the limitations of the intervention due to the brief nature of the calls and the complexity of the callers’ needs. Many staff described the importance of being caller-centered and attempting to maximize caller-centeredness within the constraints of a brief call without an established relationship (30–32).

This study takes a step toward a better understanding of how to conceptualize the quality of crisis line services and how to identify levers for strengthening quality. This is important as a 2024 national survey of 988 call center directors and crisis system leaders found that 73% of respondents felt that sufficient funding for quality monitoring would be “very important” to the success of 988 (33). While 988 centers are required to adhere to suicide risk assessment practices (34), the current study sheds light on how evidence-based interventions—as opposed to just assessments—may be better integrated into 988 calls. While the 988 policy changes might improve access to crisis lines via greater capacity and an easy-to-remember number, these changes also have the potential to increase the quality of services through systematic oversight standards (e.g., audit and feedback), performance measures, and incentives (e.g., training, financial incentives, reputation, and social incentives). While 988 centers must adhere to quality standards set forth by Vibrant Emotional Health, the national administrator of 988, Vibrant has the ability to operationalize accountability metrics that align with standards. However, as this study revealed, using Safety Planning may not sufficiently address every caller’s needs or work well with every caller. The ease of using Safety Planning also varies by factors outside the control of crisis counselors (e.g., caller circumstances and capacity to engage), which suggests the need to consider the appropriateness and feasibility of “case-mix adjustment” for any accountability metrics that aim to compare and incentivize performance across organizations (35). Unlike services anchored around ongoing treatment that obtain detailed intake assessments, operationalizing appropriate case-mix specifications would be no easy task for crisis lines, given the anonymity of callers and the limited amount of information collected about them during such brief encounters.

The quality of crisis line services is multidimensional, such that the use of Safety Planning is just one component of the service that might contribute to its utility and the caller’s experience. Indeed, a discrete individual-level intervention like Safety Planning does not directly address many callers’ needs, which could be deeply structural (e.g., employment and income) and the result of complex health and relational needs. The former category of needs cannot be addressed fully by specialty mental healthcare interventions, let alone a 15-minute phone call. While crisis lines might be able to provide connections and referrals to both healthcare and social services, implementation of these referral mechanisms is limited (36). Even if robustly implemented, they link people with services that are unlikely to significantly alter their circumstances. Staff in our study described some callers, who may be strong candidates for specialty mental health treatment, such as outpatient therapy, as left without sufficient psychological support from their interaction with the 988 crisis counselor. Moreover, being able to link a caller to healthcare services successfully is also dependent on several factors, including insurance coverage, location, transportation, employment status, income, and provider availability. Even if a connection is made, the caller may not be connected with the right provider. The fit of available providers with individual needs across various dimensions (e.g., cultural humility, expertise, experience, style, location, payment, virtual vs. in-person) is a crucial factor. Staff in our study described the need to accept the limited scope of impact that a crisis line may have. Appreciating this limited scope would also be needed when considering how to operationalize quality metrics and when imagining any potential “ideal” role that crisis lines play in society alongside other prevention and intervention strategies.

### Limitations and Future Research

While this study provides novel insights into the implementation of Safety Planning at 988 centers, the generalizability of this study is limited by the focus on crisis centers in one state, Missouri. While we interviewed staff and leadership from all crisis centers in Missouri, a major strength of the study, Missouri has also been shown to be a leader in crisis line metrics; further research should explore whether additional barriers to implementation of Safety Planning are present in states that were ranked lower on 988 performance metrics. Furthermore, both the generalizability and the nature of our understanding of Safety Planning implementation among 988 centers are limited by our study’s focus on staff perspectives.

Understanding how callers perceive Safety Planning and what comprises the “quality” of crisis lines is an important empirical question to pursue. Societal and contextual factors (e.g., political and financial factors) are likely to affect how safety planning is perceived and implemented in various environments. As such, additional work is needed to explore these questions using multiple methods and data sources (e.g., observational data, silent listening audit data, quantitative measures of attitudes, knowledge, barriers, and facilitators).

Future research is needed to understand how to best measure care quality on 988 crisis lines for both research and accountability purposes, enabling a greater understanding of how quality varies across staff, organizations, and callers, and how to leverage such measures within accountability schemes. There is a need to identify how to strike the right balance between standardized measurement while leaving enough flexibility and discretion for frontline counselors to implement person-centered care and use their experiential expertise. Additional research could also help illuminate promising approaches to effectively connecting callers with the necessary services.

## Conclusion

Safety Planning is viewed positively by 988 crisis line staff in Missouri, which is facilitated by organizational supports (e.g., culture, training, quality monitoring, feedback); however, there are barriers to its utility and impact. When implemented among 988 crisis lines, Safety Planning operates as a discrete intervention during a brief period, all without being physically co-located or being able to see the caller. Additional work is needed to identify effective ways that 988 crisis lines can triage callers to address their complex social and economic needs and circumstances. However, the fundamental solutions to those complex needs require significant social and health policy reform. There is a need to clarify the conceptualization of quality on crisis lines, including the use of Safety Planning and other evidence-based practices, and to examine how these quality dimensions vary within and across crisis line organizations. Answers to these questions will become increasingly relevant as the 988 crisis line matures into a more effective means of addressing the behavioral health crisis.

## Figures and Tables

**Table 1:** Sample characteristics

	Survey Sample		Interviews	
	(N = 97)		(N = 28)	
	n	%	n	%
Age				
18–34	45	46.4%	16	57.1%
35–54	33	34.0%	7	25.0%
55 +	19	19.6%	5	17.9%
Gender				
Male	11	11.3%	4	14.3%
Female	83	85.6%	24	85.7%
Non-binary	3	3.1%		
Race				
White	71	73.20%	21	75%
Black or African American	20	20.62%	6	17.86%
American Indian or Alaska Native	3	3.09%	1	3.57%
Asian	3	3.09%	1	3.57%
Native Hawaiian or Other Pacific Islander	0	0.00%	0	0
Other	0	0.00%	0	0
Ethnicity				
Hispanic/Latinx	7	7.22%	1	3.57%
Non- Hispanic/Latinx	90	92.78%	27	96.43%
Education				
4-Year Degree Only	27	27.80%	9	32.10%
Graduate-Level Degree	70	72.20%	19	67.90%
Role				
Crisis-line counselor	77	79.4%	21	75.0%
Leadership	20	20.6%	7	25.0%
Years of Professional Experience				
0–1 year	29	29.9%	13	46.4%
2–4 years	32	33.0%	9	32.1%
5–10 years	16	16.5%	3	10.7%
> 10 years	20	20.6%	3	10.7%
Years in Current Role				
0–1 year	41	42.3%	14	50.0%
2–4 years	31	32.0%	7	25.0%
5–10 years	16	16.5%	6	21.4%
> 10 years	9	9.3%	1	3.6%
State Licensed				
Yes	32	33.0%	6	21.4%
In the process	25	25.8%	6	21.4%
No	40	41.2%	16	57.1%
Full-Time Employment	59	60.8%	15	53.6%

Note: N = total number of respondents per sample. Percentages may not total 100 % due to rounding

**Table 2:** Characteristics associated with attitudes towards safety planning, n = 90

	Appropriateness of Safety Planning				Feasibility of Safety Planning				Acceptability of Safety Planning			
	Coef	*p*	95% CI		Coef	*p*	95% CI		Coef	*p*	95% CI	
Counselor (Ref = Leadership)	−0.01		−0.36	0.35	−0.15		−0.52	0.22	−0.16		−0.54	0.21
Full Time Employment (Ref = Part-Time Employment)	0.22		−0.07	0.51	0.05		−0.25	0.35	0.03		−0.28	0.33
Years of Employment	0.07		−0.03	0.18	0.04		−0.07	0.14	0.08		−0.03	0.19
Graduate-Level Education (Ref = Undergraduate)	−0.36	*	−0.68	−0.05	−0.35	*	−0.68	−0.03	−0.49	***	−0.82	−0.16
Licensed (Ref = Not Licensed)	0.21		−0.1	0.51	0.22		−0.09	0.53	0.07		−0.24	0.39
Years of Professional Experience	0.07		−0.02	0.16	0.08		−0.01	0.17	0.08		−0.01	0.18

Notes:

* = *p*<0.05;

*** = *p*<0.00

## Data Availability

Given the sensitive nature of the data collected in this study, we are unable to share individual-level responses to the survey or interviews. The interview guide and survey questions are available from the corresponding author upon reasonable request.

## Abbreviations

IAM: Intervention Appropriateness Measure
FIM: Feasibility of Intervention Measure
AIM: Acceptability of Intervention Measure
CFIR: The Consolidated Framework for Implementation Research
